# Cellular Senescence in Adrenocortical Biology and Its Disorders

**DOI:** 10.3390/cells10123474

**Published:** 2021-12-09

**Authors:** Xin Gao, Faping Li, Bin Liu, Yuxiong Wang, Yishu Wang, Honglan Zhou

**Affiliations:** 1 Department of Urology, The First Hospital of Jilin University, Changchun 130021, China; gaoxin0222@jlu.edu.cn (X.G.); lifp18@mails.jlu.edu.cn (F.L.); liubin20@mails.jlu.edu.cn (B.L.); wyx21@mails.jlu.edu.cn (Y.W.); 2 Key Laboratory of Pathobiology, Ministry of Education, Jilin University, Changchun 130021, China

**Keywords:** cellular senescence, adrenal cortex, compact and clear cells, aging

## Abstract

Cellular senescence is considered a physiological process along with aging and has recently been reported to be involved in the pathogenesis of many age-related disorders. Cellular senescence was first found in human fibroblasts and gradually explored in many other organs, including endocrine organs. The adrenal cortex is essential for the maintenance of blood volume, carbohydrate metabolism, reaction to stress and the development of sexual characteristics. Recently, the adrenal cortex was reported to harbor some obvious age-dependent features. For instance, the circulating levels of aldosterone and adrenal androgen gradually descend, whereas those of cortisol increase with aging. The detailed mechanisms have remained unknown, but cellular senescence was considered to play an essential role in age-related changes of the adrenal cortex. Recent studies have demonstrated that the senescent phenotype of zona glomerulosa (ZG) acts in association with reduced aldosterone production in both physiological and pathological aldosterone-producing cells, whereas senescent cortical-producing cells seemed not to have a suppressed cortisol-producing ability. In addition, accumulated lipofuscin formation, telomere shortening and cellular atrophy in zona reticularis cells during aging may account for the age-dependent decline in adrenal androgen levels. In adrenocortical disorders, including both aldosterone-producing adenoma (APA) and cortisol-producing adenoma (CPA), different cellular subtypes of tumor cells presented divergent senescent phenotypes, whereby compact cells in both APA and CPA harbored more senescent phenotypes than clear cells. Autonomous cortisol production from CPA reinforced a local cellular senescence that was more severe than that in APA. Adrenocortical carcinoma (ACC) was also reported to harbor oncogene-induced senescence, which compensatorily follows carcinogenesis and tumor progress. Adrenocortical steroids can induce not only a local senescence but also a periphery senescence in many other tissues. Therefore, herein, we systemically review the recent advances related to cellular senescence in adrenocortical biology and its associated disorders.

## 1. Introduction

Aging is a natural process accompanied by physiological degeneration of cellular function. Although the detailed mechanism of aging has remained unclarified, aged tissues feature an accumulation of cellular senescence characterized by a permanent arrest of the cell cycle. The previous conception of cellular senescence was that it is the consequence of telomere erosion due to limited divisions, termed replicative senescence [1,2]. Subsequently, researchers have demonstrated another type of cellular senescence induced by various stresses, such as oxidative stress and genomic damage [3]. Oncogene-induced senescence was also reported to serve as a protective pathway against carcinogenesis in many cancers [4]. Recently, the accumulation of cellular senescence was reported to have an impact on the pathogenesis of many age-related diseases, especially in endocrinological diseases.

The adrenal cortex is an endocrine organ that secretes many essential hormones, including aldosterone, cortisol and adrenal androgen. These steroids play pivotal roles in the balance of electrolytes and blood volume, carbohydrate metabolism and the development of sexual characteristics. The adrenal cortex is histologically classified into three distinctive layers—the zona glomerulosa (ZG), zona fasciculata (ZF) and zona reticularis (ZR). Along with deep exploration in the adrenal field, an age-dependent atrophy of the adrenal cortex has been demonstrated, especially that of the ZR layer [5,6,7,8,9,10]. In adrenocortical disorders, cortisol-producing adenomas (CPAs) were reported to produce excessive adrenal steroids, which resulted in in situ or local cellular senescence and more senescent phenotype being harbored than aldosterone-producing adenomas (APAs) [11,12,13]. Both APAs and CPAs were demonstrated to consist of two cellular subtypes, namely compact and clear tumor cells, which may harbor different senescent phenotypes reflecting intratumoral heterogeneity [11,12,14]. In addition to the in situ or local effect of adrenal steroids, they were also reported to induce a distant cellular senescence in other tissues such as the brain, kidneys, lungs, liver, bone and colon, as well as in some cancers [14,15,16,17,18]. Therefore, we herein systemically reviewed the recent advances related to the crosstalk between cellular senescence and adrenal biology, which has never been summarized before, in order to better understand the role of cellular senescence in the pathogenesis of disorders of the adrenal cortex and other organs.

### 1.1. What Is Cellular Senescence?

**Figure 1:**
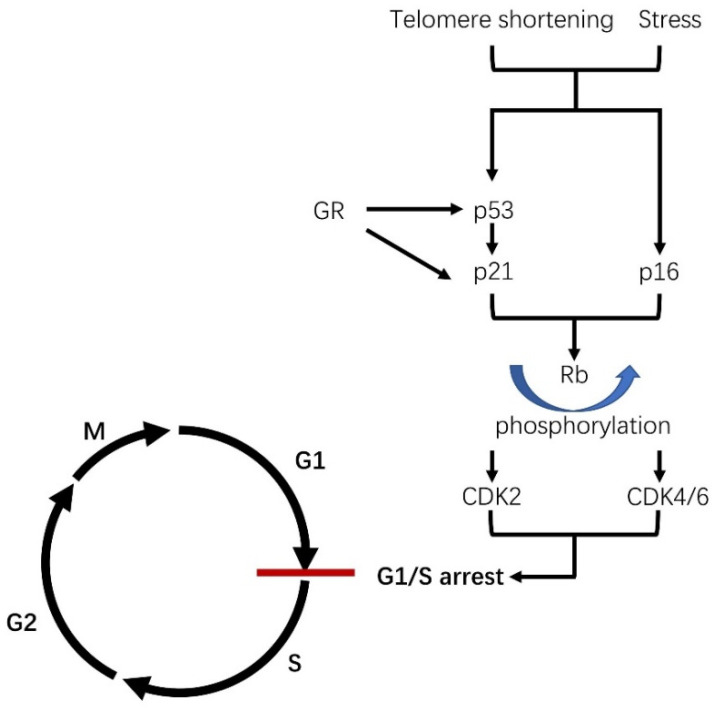
The molecular pathway of cellular senescence. The infinite cell division and stressful stimuli can finally induce a replicative senescence and stress-induced senescence, respectively. Both of those two factors activate the transcription of p53 through a DNA damage response (DDR) which subsequently stimulates that of p21. On the other hand, telomere shortening and stress can also initiate the transcription of p16. Both activation of p16 and p21 then induces the phosphorylation of the retinoblastoma (Rb) protein which induces an increased expression of cyclin-dependent kinase 2 (CDK2) or CDK4/6 resulting in cell cycle arrest of the G1/S phase. Adrenocortical steroids, such as aldosterone, cortisol and adrenal androgen, were recently reported to induce cellular senescence. Cortisol can bind to glucocorticoid receptors (GR) leading to an increase in p53 transcription under the involvement of glucocorticoid response elements (GRE) [30]. GR can also mediate p21 transcription in a p53-inpendent manner though GRE or a CCAAT/enhancer binding protein (C/EBP) [30,31]. However, the mechanisms of aldosterone and adrenal androgen in the development of cellular senescence has remained unknown.

“Senescence” is usually used interchangeably with the term “aging” to describe a decline in cellular or organic function. Several years ago, the phenomenon of cellular senescence was firstly proposed by Hayflick and Moorehead in their in vitro human fibroblast study [1]. Their great observation demonstrated that normal cells could only undergo a limited number of cell divisions in human fibroblasts, usually called replicative senescence [1,2]. Replicative senescence was later demonstrated in other cells, and the cells under the status of replicative senescence remain alive but do not divide [19]. Along with the discovery of telomeres, replicative senescence was demonstrated to cease their proliferation by telomere erosion. The gradually shortened telomere length can trigger a DNA damage response (DDR) and the ataxia-telangiectasia-mutated (ATM) kinase [20]. DDR subsequently initiates p53 transcription, which further activates its downstream regulator p21 [21,22]. In other words, shortened telomeres can also induce activation of p16 transcription, although the detailed mechanisms are not well understood for p53/p21 [23]. Both complex pathways have respective upstream regulators, downstream effectors and crosstalk between them [22,24]. In other words, the p16-induced senescent pathway imposes an irreversible arrest that is independent of the p53/p21-mediated pathways. Both p16 and p21 can induce the phosphorylation of retinoblastoma (Rb) protein, which induces an increased expression of cyclin-dependent kinase 2 (CDK2) or CDK4/6, resulting in cell cycle arrest of the G1/S phase (Figure 1). Senescent cells usually exhibit morphological changes such as an enlarged and more flattened morphology, accumulation of lysosomes and mitochondria and nuclear changes [25]. Although telomere shortening is well known and accepted to induce cellular senescence and cell cycle arrest, the detailed mechanisms still require further investigation. Later, in addition to replicative senescence, another type of senescence characterized by a rapid process was uncovered, which acts in a telomere-independent manner [3]. Some stressors such as reactive oxidative stress (ROS), DNA damage, irradiation and chemotherapy were also reported to induce this kind of cellular senescence [26]. The stress-induced senescence sometimes harbors the same overlap hallmarks as that of replicative senescence and can also cause the proliferation arrest via the DNA damage response (DDR) within several hours or days, in contrast to the several weeks needed for replicative senescence (Figure 1) [3]. After the activation of DDR, elevated expression levels of p16 and p21 will block the cell cycle in the same manner as that of replicative senescence [3,4]. For instance, ROS can be promoted by many stimuli, including tobacco smoking, UV radiation and inflammation, as a result of mitochondrial dysfunction [27,28]. The aberrant accumulation of ROS can cause damage that triggers the DDR pathway. In addition, stressors such as ROS are sometimes independent of the DDR pathway and somehow immediately stimulate the p16 pathway [29]. The aberrant accumulation of either type of senescent phenotype can negatively affect cellular regeneration and inflammatory recruitment, both of which are involved in age-related disease and cancer. However, some of the characteristics of permanent cell cycle arrest in senescent cells also protect tissues against tumorigenesis and aberrant hyperplasia.

### 1.2. Senescence-Associated Secretory Phenotype (SASP)

It should be noted that senescent cells do not die but are resistant to apoptotic cell death and remain alive with increased secretory activity, called senescence-associated secretory phenotype (SASP). SASP is one of the most important characteristics of senescent cells, which can secrete many factors, including chemokines, cytokines and growth factors [32,33]. These factors secreted by senescent cells can not only reinforce their local senescence but also induce senescent phenotypes of neighboring cells. In addition, the SASP phenotype, with its proinflammatory cytokines and chemokines, can lead to chronic inflammation, immune system activation, fibrosis, tissue damage, progenitor cell dysfunction and angiogenesis in a paracrine or endocrine manner [26,34,35,36,37]. The SASP phenotype is the result of both replicative and stress-induced senescence, including DNA damage, telomere dysfunction and epigenomic signals mediated by the DDR protein, which is the upstream regulator of p53 [29]. The genes of *NF-kB* and C/EBPβ were also considered as the main regulatory pathways for SASP under the activation of DDR. Therefore, SASP cannot be effectively initiated by p21 or p16 activation from other mechanisms, such as mitochondrial-dysfunction-associated senescence, which may not induce activation of DDR [38].

### 1.3. The Role of Cellular Senescence in Age and Its Related Disease

In older individuals, aging occurs in parallel with many factors, such as epigenetic stress, proteotoxic stress, oxidative stress, telomere damage and DNA damage, which can inevitably cause damage to tissues and cells, resulting in cellular senescence [39]. These chronic senescent cells may be assigned to defects of the aging immune system or lack sufficient ability to attract immune cells for clearance. Therefore, aged tissue composed of senescent cells from the multiple mechanisms above will become less functional and more susceptible to diseases. There is no sufficient evidence showing the link between cellular senescence and aging, although high expression levels of senescent markers, including senescence-associated β galactosidase (SA-β-GAL) and p16, were detected in aged tissues [40,41,42,43,44,45]. However, we can still speculate that cellular senescence plays a pivotal role in the aging process, and vice versa. Due to the decline in function and increased susceptibility in senescent tissues, age-related diseases frequently occur once genetic or exogenous stressors overwhelm the normal adaptability of a cell. An aberrant expression pattern of senescent markers is frequently detected in many age-related diseases. However, it is difficult to differentiate whether senescence causes age-related diseases or is the pathological consequence. Therefore, it is better to investigate the details of senescence, which will benefit our understanding of age-related diseases. For instance, senescent smooth muscle cells (SMCs) of the arteries can induce the migration and proliferation of SMCs by secreting IL-6 and Il-8, resulting in many pulmonary vascular or cardiovascular diseases [46,47]. Increased senescent cells can also induce inflammation, tissue matrix remodeling and fibrosis in the lung tissue [48]. Moreover, an accumulation of senescent cells in aged kidneys was also reported to correlate with an age-related decline in renal function [49,50]. Collectively, aging has become one of the largest risk factors for many chronic diseases associated with cellular senescence. Notably, aging and cell senescence were also reported to play essential roles in many endocrine disorders, such as osteoporosis, type 2 diabetes mellitus and pituitary tumors frequently accompanied with the senescent phenotype [51]. Senescent cells have an increased capability for protein synthesis, as we mentioned above, possibly in association with the secretion ability of endocrine disorders. Age-related changes of adrenal steroids, including aldosterone, cortisol and adrenal androgen, were reported from the end of the last century. The role of aging and cellular senescence was proposed to be involved in both physiological and pathological adrenal metabolism, resulting in different secondary metabolic disorders.

### 1.4. Physiology in the Adrenal Cortex

**Figure 2:**
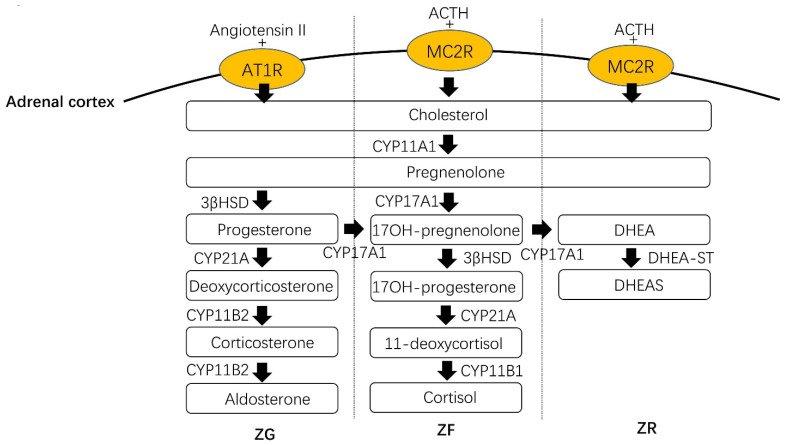
The pathway of steroids biosynthesis. The adrenal cortex receives many singling proteins by binding their receptors to stimulate steroid biosynthesis. Two key stimulators are angiotensin II (ANGII) and adrenocorticotropic hormone (ACTH) which are regulated by the renin-angiotensin-aldosterone system (RAAS) and hypothalamic-pituitary-adrenal (HPA) axis, respectively. ANGII binds to angiotensin II type 1 receptor (AT1R) to initiate aldosterone biosynthesis in ZG. Whereas ACTH binds to melanocortin receptor 2 (MC2R) resulting in the activation of cortisol and adrenal androgen biosynthesis in ZF and ZR, respectively. Cholesterol is the precursor of aldosterone, cortisol and adrenal androgen and is firstly converted to pregnenolone catalyzed by cytochrome P450 side-chain cleavage (CYP11A1) from the cytoplasm to the outer mitochondrial membrane [52,53,54]. Pregnenolone is then catalyzed into progesterone under the activation of 3β-hydroxysteroid dehydrogenase (3β-HSD) [52,53]. 21-hydroxylase (CYP21A2) then catalyze progesterone into deoxycorticosterone [55] and aldosterone synthase (CYP11B2) finally catalyzes it into aldosterone [52,53]. On the other hand, cortisol is produced in the zona fasciculata (ZF). Then, 17-alpha-hydroxylase/17,20 lyase (CYP17A1) can catalyze pregnenolone and progesterone into 17-alpha-pregnenolone and 17-alpha-progesterone, respectively. Then, 17-alpha-progesterone is converted into 11-deoxycortisol, catalyzed by CYP21A2, and finally to cortisol, catalyzed by 11β-hydroxylase (CYP11B1) [52,53,54]. Cortisol is finally biosynthesized under the activation of (CYP11B1) [52,53]. CYP17A1 can also catalyze 17-alpha-pregnenolone and 17-alpha-progesterone into dehydroepiandrosterone and androstenedione, respectively in ZR. Then they are biosynthesized to androgen by 17β-hydroxysteroid dehydrogenase type 5 (17β-HSD5) in periphery tissues [56].

The adrenal cortex is an essential endocrinological organ that synthesizes and secretes many hormones, including mineralocorticoids, glucocorticoids and adrenal androgens. These steroids contribute to balancing an equilibrium of electrolytes and blood volume, carbohydrate metabolism, reactions to stress and the development of sexual characteristics, all of which play critical roles in our daily life. Histologically, the adrenal cortex has been divided into three distinctive zones, the zona glomerulosa (ZG), zona fasciculata (ZF) and zona reticularis (ZR), which biosynthesize and secrete aldosterone, cortisol and adrenal androgen, respectively (Figure 2). Cholesterol is the precursor of these three steroids, which can be converted into different steroids step-by-step and catalyzed by different steroidogenic enzymes, such as aldosterone synthase and 11β-hydroxylase (Figure 2).

Adrenal ZG biosynthesizes and secretes aldosterone, which is an essential component of the renin-angiotensin-aldosterone system (RAAS). RAAS plays a pivotal role in the regulation of extracellular volume, sodium and potassium equilibrium and the tonus of the vascular system in the physiological status in humans [57,58,59,60]. RAAS is initiated from renin, which is in response to decreased renal perfusion. The role of renin is to catalyze angiotensinogen to angiotensin (ANG) I, with the latter being further converted to ANGII under the activation of an angiotensin-converting enzyme (ACE). ANGII is the key stimulator of aldosterone by binding to angiotensin II type 1 receptor (AT1R), expressed in adrenal ZG cells.

Adrenal ZF is regulated by the hypothalamic-pituitary-adrenal (HPA) axis responding to homeostasis, stress responses, energy metabolism and neuropsychiatric function [61]. In the HPA axis, the paraventricular nucleus of the hypothalamus (PVN) first secretes a corticotropin-releasing hormone (CRH), which then binds to its receptor 1 (CRHR1) in the anterior lobe of the pituitary gland to promote the synthesis of proopiomelanocortin (POMC), the precursor of corticotropin. The adrenocorticotropic hormone (ACTH) then stimulates cortisol biosynthesis in the adrenal ZF.

Adrenal androgens include dehydroepiandrosterone (DHEA) and dehydroepiandrosterone sulfate (DHEAS). Both of these have less affinity for the androgen receptor (AR) but can act as a precursor for potent androgens, including testosterone and dihydrotestosterone in peripheral tissues [62]. The production of DHEAS from fetal adrenal hormones promptly increases from fetal life to early postnatal life and remains at a high concentration in newborns [63,64], decreasing after this and then increasing again until adrenarche at around age 6 prior to the elevation of either estrogens or androgens associated with puberty [65,66]. Adrenal androgens are also regulated by the ACTH of HPA axis in the adrenal ZR.

## 2. Age-Related Changes in the Adrenal Cortex

The adrenal cortex originates from the intermediate mesoderm of embryological tissues, which first differentiates at 28–30 days post conception in humans [67,68,69]. It then becomes the adrenogonadal primordium, with a distinctive structure caused by the activation of steroidogenic factor 1 (SF1) [68,69]. Adrenogonadal primordium subsequently differentiates into gonadal and adrenal anlagen [69,70]. The former will differentiate into testis or ovary tissues encoded by the genes located at the XY or XX chromosome, while the latter will eventually be encapsulated and the fetal zone will emerge under the stimulation of steroid 17-alpha-hydroxylase during the last 6 weeks of gestation [71]. From the eighth week, this adrenal fetal zonation continues to differentiate into the outer definitive zone and the inner fetal zone [72]. The outer definitive zone will differentiate into the ZG and ZF soon after birth, while the inner fetal zone will subsequently differentiate into the ZR [72,73].

After the adrenal gland has matured and differentiated, it frequently renews itself to maintain its normal function via progenitor cells present in the subcapsular and capsule region [74]. Steroidogenic cells were reported to renew following the centripetal migration from the ZG to ZR [75]. In aged individuals, progenitor cells were considered to harbor less proliferative function [9]. Therefore, it has been reported that the adrenal cortex gradually becomes atrophied with abnormal hormonal function in aged adults compared with younger ones due to senescence [5,6,7,8]. In addition, adrenal nodules were reported to be accumulated during aged adrenal development, part of which is due to genetic disorders [5,6,7,8,76,77]. These nodules can be detected in subjects with either normal adrenal function, hyperaldosteronism or hypercortisolism [5,6,7,8]. During the aging process, the circulating level of adrenal steroids can fluctuate within or sometimes beyond a physiological range. These subtly altered steroid levels may chronically induce metabolic changes and the pathogenesis of age-related diseases, which is usually imperceptible for detection.

### 2.1. Age-Related Changes in Aldosterone Production

Aldosterone is an important component of the RAAS that responds to blood volume and executes its role in the kidneys. Age-dependent declines in RAAS activity have been demonstrated in normal humans in numerous studies involving many factors [78,79]. One possibility is due to the age-dependent deterioration of renal function, resulting in decreased plasma renin activity [58,80]. In addition, the response of aldosterone to angiotensin II is less sensitive, as demonstrated in older rats and women compared with younger ones [81,82]. There have also been several studies suggesting less or unchanged aldosterone secretion in older individuals [78,80,83,84,85,86]. Researchers have proposed that this less or unchanged aldosterone secretion is due to less physiological (renin-dependent) aldosterone production and more pathological (renin-independent) aldosterone production [76,87]. The less physiological aldosterone production is possibly due to the shrinkage of the ZG with decreased aldosterone synthase expression during aging, whereas the autonomous or renin-independent aldosterone production in older individuals is possibly due to the increased number of aldosterone-producing micronodules (APMs) [76]. Notably, APMs were reported to frequently harbor aldosterone-producing adenoma (APA) driving somatic mutations of various ion channels, including potassium inwardly rectifying channel subfamily J member 5 *(KCNJ5)*, calcium voltage-gated channel subunit alpha1 D *(CACNA1D)*, ATPase Na^+^/K^+^ transporting subunit alpha 1 *(ATP1A1)* and ATPase plasma membrane Ca^2+^ transporting 3 *(ATP2B3)* [11,12]. Another study also revealed the presence of numerous renin independent APMs in normal adrenal glands adjacent to APAs with autonomous aldosterone production [37]. Therefore, under genomic, aging or other uncertain factors, most APMs were recently reported to secrete aldosterone in an autonomous and renin-independent manner, considered a potential pathological process. This manner can contribute to a subclinical primary aldosteronism with normotension and normal electrolyte values accompanied by suppressed plasma renin activity with normal plasma aldosterone levels, which is frequently detected in older individuals as an age-related disease [88].

### 2.2. Age-Related Changes in Cortisol Production

The HPA axis, especially cortisol, responds to metabolism, immune reaction, cardiovascular activity and brain function. The section of cortisol in adrenaline has its own circadian rhythm, severing as an “adrenal clock” [89,90]. The circulating cortisol levels are variable due to rhythm fluctuations and stressful stimuli. The circulating cortisol level typically increases in the early morning and decreases throughout the rest of the day, finally reaching a nadir at midnight [91]. This rhythmic cortisol level can also fluctuate due to other factors, one of which is age. Some evidence has shown that the circulating level of cortisol increases with aging [92,93,94]. This elevated level of cortisol was regulated by circadian rhythmicity with an age-dependent increase in nocturnal cortisol in normal individuals, which was enhanced in older individuals with senile dementia. Increased diurnal cortisol was also detected in some studies [95]. In addition, the decreased number of glucocorticoid receptors (GRs) was reported to decrease with age, implying the involvement of the attenuated negative feedback system and resulting in high cortisol levels [96]. Studies focusing on the age-related sensitivity of the HPA axis showed conflicting results. Some studies suggested that the responsiveness of the pituitary and adrenal glands to exogenous CRH was not influenced by aging [97,98], whereas other studies demonstrated both cortisol and ACTH become more sensitive to CRH with aging [99,100,101]. It has also been reported that the elevated cortisol level was in association with age-related diseases, such as Alzheimer’s disease, and due to neuronal loss [102,103,104]. In addition, the age-related fluctuations of cortisol levels also depend on sexual dimorphism. For instance, age-related reductions in corticosteroid-binding globulins (CBGs), which bind to free plasma or activated corticosteroids and then act on their targets, were detected only in older male subjects [97]. Slightly higher salivary cortisol levels with a flattened diurnal pattern were also frequently detected in older male subjects [105]. These sexual dimorphisms of age-related fluctuations are possibly due to the effects of androgen or estrogen on the HPA axis [106].

### 2.3. Age-Related Changes in Adrenal Androgen Production

Adrenal androgens including DHEA and DHEAS are active in the phase of fetal life and early postnatal life. After this, adrenal androgens return to a low level until adrenarche. In contrast to the slight elevation of the cortisol level with aging, adrenal androgens begin to decline at a rate of 1–2% per year from the third decade, finally reaching 20–30% of the peak levels of young people by the ages 70–80 [107,108]. The decrease in adrenal androgens is considered to be due to age-dependent morphological changes of the ZR, which showed degenerative changes with aging [109]. The detailed mechanism has remained unclear because investigating DHEA and DHEAS is quite difficult due to the absence of both DHEA and DHEAS in non-primate animals. However, some researchers have raised some viewpoints that the reduction in ZR cells is the consequence of increased apoptosis activity during aging [110]. The atrophic ZR was also due to a senescent phenotype caused by aging-accumulated lipofuscin, indicating an impaired steroid metabolism capacity due to dysfunctional mitochondria [111]. The decline in adrenal androgen cannot be regulated by the negative feedback system within a stable range [112]. In addition, impaired responsiveness of DHEAS to exogenous ACTH was detected in older subjects compared with that of cortisol [113]. Furthermore, the decline in DHEAS was due to the inhibitory activity of insulin, which increased with aging, as demonstrated in older men [114].

## 3. Cellular Senescence in Normal Adrenal Cortex and Its Disorders

**Figure 3:**
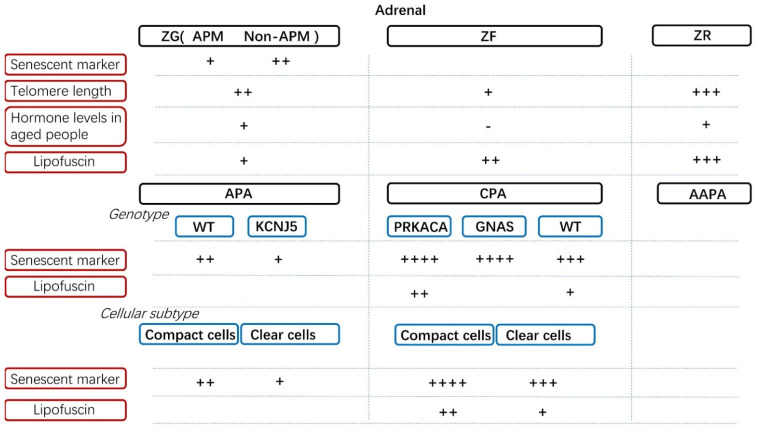
The senescent phenotype in the adrenal cortex. There was no discrepancy of senescent markers including p16 and p21 among ZG, ZF and ZR. However, aldosterone-producing micronodules (APM) harbored more abundant senescent markers than non-APM cells of ZG. The telomere length increased in the order of the ZF, ZG and ZR, whereas the lipofuscin accumulation increased in the order of the ZG, ZF and ZR. In aged people, aldosterone and adrenal androgen were reported to elevate in contrast to the decline of cortisol level. In adrenocortical disorders, CPAs were reported to harbor more abundant senescent markers than APAs, especially in *PRKACA* and *GNAS*-mutated ones. In addition, lipofuscin was more abundant in *PRKACA*-mutated CPA than wild types. Based on cellular morphology, senescent markers and lipofuscin were more abundant in compact cells than clear cells, especially in CPAs than in APAs which has not been investigated in adrenal androgen-producing adenoma (AAPA).

Many studies have revealed an intensive influence of aging on adrenocortical metabolism. The detailed mechanisms remain unknown, although cellular senescence is considered one of the factors that contributes to this age-dependent phenomenon. A divergent expression pattern of senescent markers has been discovered in the adrenal cortex, and the associated disorders have been analyzed for their relationships with hormonal activity (Figure 3) [11,115,116,117].

The adrenocortical zone is differentiated from adrenal progenitor and stem cells during the neonatal phase. This differentiation causes the appearance of three distinctive zones, namely the ZG, ZF and ZR. Previously, researchers considered that the whole ZG was reasonable for aldosterone production; however, it was recently noted that aldosterone is not biosynthesized in all ZG cells but rather in aldosterone-producing micronodules (APMs) of the ZG, which were previously called aldosterone-producing cluster cells (APCCs) [118,119]. The functions of other cells in the ZG excluding APMs have remained unknown, and those cells were demonstrated to have no aldosterone production ability due to the absence of CYP11B2 (aldosterone synthase) expression [118,119]. In contrast, cortisol and adrenal androgen are biosynthesized and secreted throughout the ZF and ZR, respectively. Due to the functional decline during the aging process caused by stressful stimuli, genomic injury and reduced differentiation and renewal ability in adrenocortical cells, normal adrenocortical cells become senescent to different extents. Based on the limited number of senescent reports in normal adrenal function from autopsy studies, ZG cells harbor more senescent markers than APMs, including p16 and p21 [11]. This suggests that normal aldosterone-producing cells require more energy with less senescence for hormonal activity than non-functional ZG cells. However, there is no difference between p16 and p21 among the ZG, ZF and ZR [11]. In addition, telomere length was reported to be longer in the order of the ZR, ZG and ZF in subjects aged 20 to 68, but no difference was seen in the younger group [115]. The telomere length of ZR cells also increased with age in the older group rather than the younger group, especially during adulthood [115]. The telomere is a non-coding repetitive DNA sequence located at the ends of chromosomes as the trigger of replicative senescence [120]. The shortest telomere length in the ZF, which is considered the most senescent layer, was possibly due to the higher amount of cortisol requirement for daily life than for other hormones. Therefore, ZF cells may need to frequently divide to maintain the high secretion level of cortisol and the wider structure of the ZF. On the other hand, the shortened telomere length is also the result of local or in situ damage by cortisol [121]. The increased telomere length of the ZR with aging is possibly due to the decline in both DHEA and DHEAs in older adults [115,122].

Adrenocortical tumors are common adrenal diseases as the result of abnormal differentiation of adrenocortical cells, which is usually caused by genetic disorders. Benign adrenocortical tumors include aldosterone-producing adenomas (APAs), cortisol-producing adenomas (CPAs) and androgen-producing adenomas. APA is the major cause of primary aldosteronism (PA), which accounts for 5–10% of all hypertensive patients [123]. The formation of APA is caused by somatic mutations of the aldosterone-driver gene, such as *KCNJ5*, *CACNA1D*, *ATP1A1* and *ATP2B3* [12]. CPA is the result of abnormal differentiation from ZF cells and is usually caused by somatic mutations of protein kinase cAMP-activated catalytic subunit alpha (*PRKACA*)*,* GNAS complex locus (*GNAS*) and catenin beta 1 (*CTNNB1*), resulting in hypercortisolism and clinical Cushing’s syndrome [106]. Cases of adrenal androgen-secreting adenoma are rare and possibly caused by the degenerative ZR. Adrenocortical carcinoma is a malignant adrenal tumor that also presents at a low rate but is always accompanied by hormone excess, including aldosterone, cortisol estrogen and androgen [124,125]. Therefore, the greatest feature of adrenal tumors is not only their occupancy and malignancy but their accompanied hormonal excess. When adrenal tumor cells were exposed to excessive steroids secreted by adrenal tumors beyond the dose they can manage, they were driven into an in situ and distant cellular senescence [11,117].

Focusing on the morphology, adrenal tumor cells are basically classified into compact and clear cells, also termed ZG- or ZF-like cells from a histological standpoint [38,39,40,41]. Based on the cellular morphology, clear cells have a low ratio of nuclear-to-cytoplasm-harboring relative abundance lipid droplets in the cytoplasm. On the other hand, compact cells have a high ratio of nuclear-to-cytoplasm cells, with an eosinophilic lipid-poor cytoplasm containing relatively abundant cell organelles, especially mitochondria. However, detailed features and functional aspects of both compact cells and clear cells have remained unclear. Recent studies showed that both compact and clear cells had different hormonal activities, representing different statuses of cellular senescence [116,117]. The different ratios of compact and clear cells in adrenal tumors also reflect intratumoral heterogeneity in terms of cellular senescence.

In addition, adrenal steroids can induce not only in situ cellular senescence, but also a distant cellular senescence in other tissues. For instance, adrenal steroids were reported to induce a senescent phenotype in human organs such as the kidneys, liver, brain, breasts and bone via either the p53/p21, p16 or SA-β-GAL pathways. Next, we will systemically review the details of the association between adrenal steroids and cellular senescence.

### 3.1. Cellular Senescence in Aldosterone-Producing Adenoma

The senescent phenotype has rarely been investigated in adrenocortical adenomas. In patients with aldosterone-producing adenomas, p21 expression is higher in the normal adrenal ZG adjacent to the APA (adjacent ZG) than the APA [11], while p16 expression is also higher in the adjacent ZG compared with the adjacent ZF, ZR and APA [11]. Furthermore, in idiopathic hyperaldosteronism (IHA) with multiple nodules, only p16 is predominantly immunolocalized in the ZG (non-aldosterone producing cells) as compared with the ZR and aldosterone-producing nodules. Those results demonstrated that CYP11B2-negative ZG cells were more senescent than other cortical cells, especially in the case of APA and IHA. In addition, aldosterone-producing cells harbored a less senescent phenotype than CYP11B2-negative ZG cells. This indicated that less cellular senescence can result in high hormonal activity of aldosterone-producing cells. The adjacent ZG under suppressed plasma renin activity (PRA) was reported to present a silent or inactive status, harboring less aldosterone-producing ability compared with the normal ZG [126]. One of the reasons for this inactive status was the high cellular senescence leading to impaired aldosterone biosynthesis. Considering the influence of the genotype, p21 expression was more abundant in wild-type APAs than in *KCNJ5*-mutated ones [11]. The senescent phenotype of wild-type APAs resulted in a smaller tumor size as a consequence of replicative arrest and inactive hormonal activity, with less aldosterone production compared with *KCNJ5*-mutated APAs [11]. As we mentioned above, the adrenocortical tumors consisted of different subtypes of adrenocortical cells, including compact cells and clear cells. These two subtypes of cells harbored divergent cellular components, resulting in intratumoral heterogeneity. A previous study demonstrated that CYP11B2 immunoreactivity positively increased with the number of clear cells in APAs, especially in *KCNJ5*-mutated APAs [127]. In both *ATP1A1*- and *CACNA1D*-mutated APAs, compact cells were the predominant subtype, although the expression pattern of p16 and p21 has not been explored in these genotypes yet [128]. Clear cells were demonstrated to express less p16 and p21 markers compared with compact cells in APAs, and this tendency also exhibited a genotype-dependent manner [11].

### 3.2. Cellular Senescence in Cortisol-Producing Adenoma

In contrast to the predominant component of clear cells in APAs, CPAs were reported to consist of divergent ratios of compact cells and clear cells [117]. In CPAs, compact cells were reported to harbor more abundant cortisol-producing steroidogenic enzymes, including CYP11B1 and CYP17A1 than clear cells [117]. Compact cells accompanied with abundant hormonal activity of CPAs were demonstrated to induce in situ cellular senescence due to p16 expression being higher in compact cells than in clear cells [117]. The senescent compact cells subsequently reduced the cell proliferation, which was consistent with the result showing that the Ki-67 labeling index was lower in compact cells, and the immunoreactivity of both CYP17A1 and p21 were inversely correlated with the tumor size of CPAs [117]. Therefore, the strong hormonal ability in CPAs can induce a replicative senescence, which seems to have no effect on hormonal biosynthesis. When we consider the influence of genotypes on the hormonal activity and cellular senescence of CPAs, *PRKACA*-mutated CPAs are associated with higher clinically autonomous cortisol production than *GNAS*-mutated and wild-type CPAs [111,122,123]. In addition, hybrid steroidogenic enzymes of both cortisol and DHEAS were reported to be detected in CPAs [129]. The key enzyme for DHEAS, dehydroepiandrosterone sulfotransferase (DHEA-ST), was also reported to be more highly expressed in hormonally active compact cells than clear cells [117]. There was a positive correlation between DHEA-ST and p21 reported in CPAs, indicating that DHEA-ST could also induce in situ cellular senescence. This autonomous DHEA-ST production was increased in *GNAS*-mutated CPAs compared with *PRKACA*-mutated and wild-type CPAs [117]. Therefore, both *PRKACA*-mutated and *GNAS*-mutated CPAs with advanced autonomous cortisol and DHEA-S production, respectively, display a more senescent phenotype with a smaller tumor size than wild-type ones.

### 3.3. Comparison of Senescence between Different Adrenal Hormone-Producing Adenoma

The effect of the senescent phenotype towards hormonal activity is divergent between APAs and CPAs. For instance, cellular senescence in CPA was detected as a consequence of excessive cortisol and DHEAS production in situ [117]. However, cellular senescence in APAs was identified as being associated with suppressed aldosterone production activity [11]. Endocrine tissues promote their hormonal production by increasing the mitochondrial function or proliferation of endocrine cells. This indicates that the cellular senescence in APAs may restrain the cell proliferation to control aldosterone production due to cellular senescence being negatively correlated with tumor size [11]. However, the senescent phenotype of mitochondria remains to be uncovered. In addition to APA, CPAs were demonstrated to harbor a more senescent phenotype but with more abundant p16 and p21 expression than APAs [13]. Under the severe status of cellular senescence, CPAs showed more intratumoral immune cell infiltration than APAs due to recruitment by SASP [13]. The severe senescent phenotype seems not to have a negative effect on cortisol production in CPAs. The features of morphology-based cellular subtypes also exhibited different patterns between APAs and CPAs. Compact and clear cells were named as such based on morphological features of the ZG and ZF. Therefore, researchers naturally believed that clear cells should inherit the function of ZG cells producing aldosterone and that compact cells are responsible for cortisol production in association with ZF cells. However, the tumor cells of APAs and CPAs exhibited a deviated phenotype from the normal zonation of the adrenal cortex, whereby clear cells in APAs harbored more aldosterone-producing ability, whereas compact cells in CPAs harbored more hormonal activity, including both cortisol and DHEAS. However, compact cells in both APAs and CPAs were demonstrated to accompany an abundant senescent phenotype, resulting in differing hormonal abilities, especially in genotype-based subtypes.

### 3.4. Cellular Senescence in Adrenocortical Carcinoma

We have discussed the characteristics of both proliferative senescence and stress-induced senescence in the former content. However, it should be noted that both kinds of senescent phenotypes maintain limited roles in carcinogenesis, which allows tumors to grow indefinitely. Oncogene-induced senescence was reported as another type of senescent phenotype that plays an important role in many cancers to restrict their progress as a compensatory pathway [4]. Adrenocortical carcinoma is a rare malignant tumor type that is frequently accompanied by poor diagnosis and is detectable for oncogene-induced senescence [124,125]. For instance, previous studies have demonstrated that ACC showed greater expression of p21 and p53 following high Ki-67 expression in comparison to adrenocortical adenomas (ACAs) [130,131]. However, telomerase activity was greater in ACCs than ACAs and significantly correlated with the size of ACCs rather than ACAs, indicating that proliferative senescence was rarely present in ACCs [132]. In a clinical study, high CDK6 expression was demonstrated to be correlated with poor prognosis in patients with ACCs. In vitro studies showed that the treatment of the CDK6 inhibitor could induce cellular senescence, resulting in antiproliferation through glycogen synthase kinase 3β (GSK3-β) and β-catenin pathways in human adrenocortical (H295R) cell lines [132]. Genetic disorders were also reported to frequently occur in the pathogenesis of ACCs, such as mutations of p53 and CTNNB1 [124]. The gene of p53 plays a central role in responding to genotoxic stress and oncogene activation via cell cycle arrest. In addition, the somatic mutation of p53 accounts for 20–70% of ACCs and the germline mutation of p53 was identified in pediatric ACCs at a rate of 50–80% [133]. Although the status of p53 mutation did not exhibit any prognostic relevance, an increased aberrant p53 expression was shown in association with poor diagnosis following activated oncogenesis [134,135]. It is well known that p53 also actives apoptosis pathways. However, the mechanism detailing how p53 chooses between cell senescence and apoptosis has remained unclear. However, it has been suggested that apoptosis is a consequence of overwhelming stress, whereas cellular senescence responds to less stress or damage [136]. In addition, the activation of p53 can be detected in both senescent and apoptotic cases [137]. Wnt signaling is critical for cell differentiation, morphogenesis and tumorigenesis, and is kept at low levels in normal cells [138]. Therefore, the mutation of *CTNNB1* causes an accumulation of β-catenin in the cytoplasm and subsequently activates downstream effectors, resulting in infinite proliferation [139]. Loss of β-catenin in H295R cells was demonstrated to play an antiproliferation role compared with the control cells [139]. In addition, the transplantation of β-catenin-downregulated ACC cells in mice reduced malignant behavior in tumors via increased p21 expression compared with that of control cells [139]. Clinically, *CTNNB1*-mutated ACCs were reported to harbor poor overall survival and are characterized by immune exclusion [140]. Immune cells including CD3, CD4, CD8 and FoxP3-positive cells were predominantly identified in ACC patients in association with patient prognosis, although an association with SASP remains to be clarified [141]. ACC is also characterized by hormonal excess, which leads to many secondary metabolic disorders, such as hypertension or Cushing’s syndrome. The local or in situ effects of ACC-producing hormones on the cellular senescence of ACC have never been explored. In CPAs, the overproduction of cortisol has a locally antiproliferative effect, as we mentioned above. However, excessive cortisol production was considered a negative prognostic marker in both advanced and localized ACCs, which is inconsistent with the result for CPAs [142]. The detailed mechanisms remain unknown, but we guess that the overproduction of cortisol can suppress local immune cells in ACCs. The effect of suppressed immunocytes on ACC progress overwhelms that of cortisol-induced local cellular senescence, resulting in an aggressive state with poor diagnosis [141].

### 3.5. The Role of Lipofuscin in the Adrenal Gland and Its Related Disorders

Along with the increase in age, many deposited pigments characterized by brown-yellow and autofluorescence were accumulated in cells [143]. These deposited granules were named lipofuscin, which were composed of a protein and lipid and considered a senescent marker [144]. Lipofuscin was reported to be identified in cardiac myocytes, hepatocytes and brain neurons, as well as the adrenal cortex, and the accumulation of lipofuscin was negatively correlated with longevity [145,146,147,148]. Numerous mechanisms are involved in the development of lipofuscin, with the well-accepted one being that lipofuscin is the residue caused by the intracellular accumulation of lysosomal degradation that cannot be cleared by exocytosis [144]. In the rat adrenal cortex, an increased quantification of lipofuscin was detected in the order of the ZG, ZF and ZR [149]. The same result was also demonstrated in the human adrenal cortex, indicating that ZR harbored a more senescent phenotype during the aging process [148,150]. Additionally, in aged individuals, the accumulation of lipofuscin was observed in association with a decline in DHEAS production, which was consistent with the age-dependent decline in adrenal androgen [148]. When the treatment of ACTH was performed in normal rats aged 23 months, a diminished amount of lipofuscin was demonstrated in the rat adrenal cortex, possibly indicating that a less senescent phenotype was necessary for increased hormonal activity and proliferation [151]. A pathologically abnormal deposition of pigments accounts for certain kinds of adrenal disorders accompanied with subclinical or overt hypertension or Cushing’s syndrome [152]. The pigmented disorders are histologically classified into two subtypes, namely primary pigmented nodular adrenocortical disease (PPNAD) and adrenal black adenomas. The detailed pathogenesis of the above disorders remains unknown, although the somatic mutation of protein kinase cAMP-dependent type I regulatory subunit alpha (PRKAR1A) was identified in PPNAD [153]. In addition, they were also reported to frequently consist of cortisol-producing cells rather than other adrenocortical cells manifesting as subclinical or overt Cushing’s syndrome [152]. PPNAD is usually characterized by normal- or small-sized adrenal glands harboring numerous brown or black nodules surrounded by atrophic adrenocortical cells [153]. The surrounding atrophic adrenocortical cells are possibly the result of suppressed ACTH levels [153]. An adrenal black adenoma is a benign tumor composed of compact cells containing multiple yellow-brown pigments characterized by lipofuscin [154,155]. Both adrenal black adenomas and PPNAD are considered senescent phenotypes due to their numerous pigmented granules and the compact cell components. Furthermore, excessive cortisol production may further induce local cellular senescence, although this still needs to be confirmed. The ultrastructural study revealed a severe impairment of mitochondria in adrenal black adenomas, which possibly results in senescence against hormonal secretions [154]. Lipofuscin was also more accumulated in CPAs than APAs, especially in *PRKACA*-mutated cells, which was consistent with the fact, mentioned above, that *PRKACA*- and *GNAS*-mutated CPAs harbored more senescent markers compared with wild-type ones and APAs. In androgen-producing adrenocortical carcinomas, lipofuscin was detectable in compact tumor cells, revealing the close association of lipofuscin and adrenal androgen [156].

### 3.6. The Role of Cellular Senescence in Adrenal Physiology during Aging

**Figure 4:**
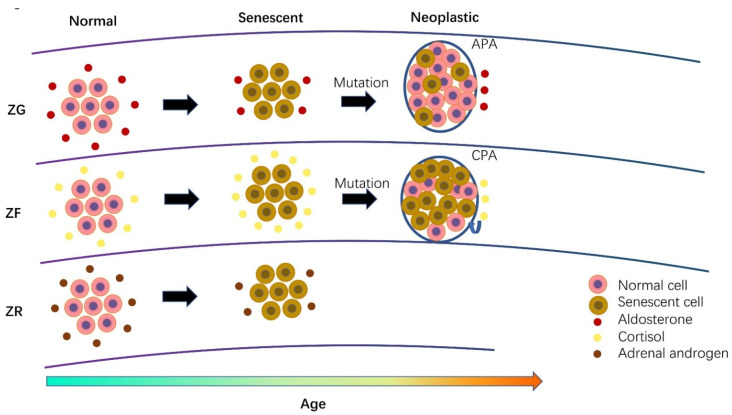
The role of cellular senescence in hormonal function of the adrenal cortex during aging. ZG, ZF and ZR cells have similar senescent phenotypes during aging. However, the effect of senescence on hormonal function is different among these three cells. We proposed that cellular senescence may suppress aldosterone and adrenal androgen production but not cortisol production. Therefore, aldosterone and adrenal androgen production are gradually reduced, whereas cortisol production tends to gradually increase during aging. When a neoplastic nodule develops due to genetic disorders, both aldosterone-producing adenoma (APA) and cortisol-producing adenoma (CPA) can autonomously biosynthesize and secrete aldosterone and cortisol. This autonomous overproduction of steroids also reinforces a local or in situ senescent phenotype resulting in a more senescent phenotype in CPA than that in APA. The role of cellular senescence in adrenal androgen producing adenoma has rarely been explored and remained unknown.

Aging is an important factor that induces chronic changes in both the physiological function and structure of the human adrenal gland (Figure 4). Adrenocortical cells become atrophied during aging, resulting in an altered width and hormonal production. Slightly decreased circulating aldosterone and slightly increased circulating cortisol levels were detectable without any clinical manifestation. Adrenal androgen production also gradually reduced after the period of puberty. All of these phenomena above can be considered the consequences of cellular senescence. Accumulated cellular senescence in the aging adrenal ZG may suppress aldosterone-producing ability to some extent [11]. However, accumulated senescence in aging cortisol-producing cells of the ZF does not have any negative impact on cortisol production or even stimulate secretory ability [117]. The slightly and physiologically increased cortisol levels seem not to aggravate local senescence, as there were no significant differences in p16 and p21 markers among the ZG, ZF and ZR [11]. In addition, ZR cells have the shortest telomere length as compared with ZG and ZF cells, demonstrating a reduced renewal in ZR cells [115]. The presence of much more lipofuscin was also reported in ZR cells as compared with ZF and ZG cells [148,150]. Lipofuscin contains undegradable components, the accumulation of which was reported as the result of impaired mitochondrial function [111]. Therefore, we can assume that both reduced cellular renewal and impaired mitochondrial function lead to an age-dependent decline in adrenal androgen after the third decade.

## 4. Adrenocortical Steroids Induce Distant Cellular Senescence in Other Tissues

The adrenal gland is an endocrine organ that can connect with other organs by secreting hormones. Under the action of circulating adrenocortical steroids, many peripheral tissues are involved in cellular senescence, as demonstrated by many in vivo and in vitro studies. Aberrant production of adrenocortical steroids can also induce oxidative stress and genomic damage, including telomere shortening, both of which play important roles in the development of cellular senescence [121,157,158,159]. Adrenocortical steroids including aldosterone, cortisol and adrenal androgens execute their roles in peripheral tissues through their receptors, which subsequently initiate a direct or indirect stimulation of the p53–p21 pathway or the p16 pathway in many tissues, eventually resulting in cellular senescence [30]. Cortisol was reported to bind to the glucocorticoid receptor (GR) and directly induce the activation of p53 through glucocorticoid response elements (GRE) [30]. Activated p53 subsequently stimulates p21 transcription, resulting in cell cycle arrest via the inhibition of cyclin-dependent kinase (CDK) 2 or CDK4/6 (Figure 1) [50]. The direct molecular pathway between MR and cellular senescence has not been demonstrated yet, although an accumulation of oxidative stress under excessive aldosterone action revealed an indirect pathway between them [157,158]. In addition, there was no obvious evidence that adrenocortical hormones induced either stress-induced senescence or replicative senescence. Based on present evidence, the senescent phenotype caused by adrenocortical hormones was mostly considered stress-induced senescence, whereas the replicative one tends to be a natural process dependent on telomere erosion which has a little role in hormone effects.

### 4.1. Aldosterone and Glucocorticoid-Induced Cellular Senescence in the Kidneys

Aldosterone physiologically binds to the mineralocorticoid receptor (MR) in the role of regulating the equilibrium of electrolytes and blood volume. Abnormal elevated aldosterone levels can induce renal injury, chronic kidney disease and renal fibrosis [160]. Additionally, aldosterone actions are also involved in cellular senescence, which damages renal function [14]. Increased p53 and p21 in both mRNA and protein levels were detected in aldosterone-infused rats compared with vehicle-infused rats, and this stimulation was attenuated by eplerenone, an MR blocker [14]. SA-β-GAL activity was also examined and showed a parallelly increased level, especially in proximal tubules rather than the glomeruli of aldosterone-infused rats as compared with control rats [14]. These phenomena of aldosterone-induced senescence were further demonstrated in human proximal tubular cells [14]. In addition, aldosterone-induced senescence plays an inhibited role in renal injury recovery because aldosterone can delay wound healing in human proximal tubular cells, which could be suppressed by transfection with siRNA for p21 [14]. Aldosterone-induced renal cell senescence has never been investigated in humans, although some studies have demonstrated the presence of cellular senescence in kidney disease, including CKD, kidney transplantation, autosomal-dominant polycystic kidney disease and acute kidney injury causing renal dysfunction [50,161].

The accumulation of senescent marker p21 has been previously reported in acute kidney injury, the detailed mechanisms of which remain unclear [162,163]. This senescent phenotype in acute kidney injury has been recently demonstrated to be mediated by glucocorticoid [164]. Increased transcription of p21 was detected in rat models with acute kidney injury [164]. This increased p21 expression can be normalized by treatment of the GR antagonist in rat models with acute kidney injury, whereas no difference was detected in normal rats with the same treatment [164]. In addition, dexamethasone could also induce an increase in p21 in normal renal cortical cells of rats at both mRNA and protein levels [164]. Glucocorticoid is also administrated to ameliorate mesangial proliferative glomerulonephritis, indicating its role in replicative senescence [165]. Suppressed expression of CDK2 and CDK4 was detected in mesangial cells treated by dexamethasone following increased p21 levels, which resulted in cell cycle arrest within the S and G2/M phases [165]. These results were also demonstrated in a rat study in vivo [165]. Prednisolone administration could significantly ameliorate mesangial proliferative glomerulonephritis in rats following increased protein levels of p21 and decreased levels of CDK2 and CDK4 [165]. The remission of proteinuria was also detected in mesangial proliferative glomerulonephritis rats treated with prednisolone, compared with the untreated group [165].

### 4.2. Aldosterone-Induced Cellular Senescence in Vascular Smooth Muscle Cells

Since the presence of MR was first detected in the vasculature by Sasano et al., in 1986, research on the MR-mediated effects on the vasculature, including vascular smooth muscle cells (VSMC), has been ongoing [166]. RAAS components, such as ANGII and aldosterone, were frequently reported to induce oxidative stress resulting in vascular damage, such as vascular remodeling and atherosclerosis [167,168,169]. Recently, aldosterone- and ANGII-mediated senescence in VSMCs contributing to vascular damage has also been reported. In VSMCs, ANGII and aldosterone can induce an increased number of SA-β-GAL-positive cells in a time-dependent manner [170,171]. In addition, increased expression of p21, p53 and p16 was also detected in VSMCs treated by ANGII and aldosterone [170,171]. This stimulation could be blocked by selective angiotensin type 1 receptor (AT1R) blocker and MR antagonist [170]. The increased transcription of p21 and p53 by ANGII was also demonstrated in mice models [171]. In the same model, ANGII treatment strikingly induced the expression of proinflammatory cytokines such as IL-1β and IL-6 through a p21-dependent pathway, which may contribute to the induction of atherosclerosis [171]. There are some other factors involved in cell senescence, such as the Ras gene and oxidative stress [172,173]. Ki-ras2A was demonstrated to be stimulated by ANGII and aldosterone, contributing to cellular senescence, which can be attenuated by the knockdown of the Ki-ras2A gene [173]. NADPH oxidase activity can be stimulated by ANGII and aldosterone treatment in VSMCs, inducing cellular senescence [173]. In pulmonary vascular cells, aldosterone has been demonstrated to induce cellular senescence and inhibit the viability of endothelial progenitor cells, which prevents their differentiation into new blood vessels [174].

### 4.3. Glucocorticoid-Induced Cellular Senescence in Hepatoma Cells

Glucocorticoid can also cause an inhibition of cell proliferation through the p21 pathway in hepatoma cells. In rat hepatoma cells, stimulated p21 expression was identified under the treatment of dexamethasone, resulting in cell cycle arrest within the G1 phase [17]. In addition, cell cycle components including CDK2, CDK4, CDK6 and cyclin D1 were further demonstrated to increase in the same cell lines treated by dexamethasone [17]. Additionally, p21 is well known as a downstream regulator of p53 to control cell proliferation. However, in rat hepatoma cells, the response of p21 to GR was demonstrated in a specific p53-independent manner [17]. Researchers have demonstrated a novel pathway between p21 and GR without p53 activation [31]. In the p21 gene, there is a DNA-binding site for the transcription factor CCAAT/enhancer binging protein-α (C/EBPα), which can be mediated by the GR response [31]. The increased C/EBPα expression was demonstrated in rat hepatoma and normal live cells under the administration of dexamethasone [31]. Moreover, in rat hepatoma cells lacking the C/EBPα gene, dexamethasone treatment was unable to activate p21 transcription, indicating that the regulation of C/EBPα was necessary for GR-mediated p21 transcription [31]. However, the role of GR-mediated cellular senescence has been rarely explored in human hepatoma studies, although one study reported weakly activated p21 expression induced by dexamethasone, which still needs to be investigated further [175].

### 4.4. Glucocorticoid-Induced Cellular Senescence in the Development of Fetal Lungs and Lung Cancers

Glucocorticoids are well known to play pivotal roles in the maturation of fetal lungs before birth [176,177,178]. The severe lung cellular hyperplasia and immature lung morphology were detected in germline GR-knockout mice, resulting in death caused by respiratory failure [16]. Despite the role of glucocorticoids in the development of fetal lung tissues, the details of glucocorticoid signaling in fetal lungs are poorly understood. However, a recent study demonstrated the importance of GR-induced cellular senescence in the development of fetal lung tissues in rats to avoid hyperplasia [16]. The results demonstrated that p21 expression decreased in GR knockdown rats, resulting in high rates of proliferation and cellular hyperplasia compared with the control rats [16]. Considering the role of glucocorticoid-induced cellular senescence in lung tissues, glucocorticoid treatment was also reported to alleviate proliferative progression of lung cancers [179,180,181,182]. GR was reported to immunolocalize in many kinds of primary lung cancer, including squamous-cell carcinomas, adenocarcinomas, large-cell carcinomas, carcinoids, bronchoalveolar-cell carcinoma and small-cell lung cancer [182]. In non-small cell lung cancer (NSCLC) cell lines, the treatment of dexamethasone greatly suppressed cell proliferation involving the activation of p21 [180]. Inhibition of CDK2 and CDK4 expression was also detected under dexamethasone treatment following the activation of p21 and resulting in G1 cycle arrest [180]. In addition, dexamethasone was demonstrated to induce SA-β-GAL activity and the loss of Ki-67 expression in lung adenocarcinoma cell lines [179]. However, p16 activity was unchanged following dexamethasone treatment in lung cancer cell lines [179,180]. In a human in vivo study, patients who harbored high expression levels of GR showed clinically longer durations of progression-free survival (8.0 vs. 5.6 months) and overall survival (18.1 vs. 10.2 months) compared with patients with low or no expression of GR [181].

### 4.5. Glucocorticoid-Induced Cellular Senescence in Neural Cells

Glucocorticoids also play pivotal roles in the development and maintenance of brain structures, especially the hippocampus. In the fetal phase, the human placenta actively expresses the 11β-hydroxysteroid dehydrogenase 2 (11β-HSD2) enzyme, whose role is to inactivate cortisol into cortisone, which cannot bind to GR to protect high maternal glucocorticoid levels during pregnancy [183,184,185]. Evidence from animal experiments has demonstrated that intrauterine exposure to excessive glucocorticoids has negative impacts on brain development, resulting in impairment of social interaction and deficits in memory, triggering anxiety-like behavior [186,187,188]. The action of glucocorticoids was also reported to attenuate neurogenesis due to proliferative senescence, resulting in hippocampal cell loss [189,190,191,192]. Dexamethasone was demonstrated to stimulate the activation of p53 and p21 and ultimately induced cell cycle arrest of the G1 phase in human neural cell lines [15]. Adrenalectomy in the mid-life of rats alleviates the impaired hippocampal degradation and cognition in association with low circulating glucocorticoid levels. In Cushing’s syndrome with chronic glucocorticoid excess, patients frequently harbored structural and functional changes in the central nervous system displaying depression, anxiety, panic disorders and neurocognitive impairment [193]. These symptoms were attributed to brain atrophy, possibly due to GR-mediated antiproliferation [193,194]. In addition, age-dependent elevation of cortisol levels was reported to be accompanied by many aging-related mental diseases, including depression, cognitive deficits and Alzheimer’s disease [195,196,197]. Another study demonstrated that impaired explicit memory with a 14% reduction in hippocampal volume was detected in aging humans with increasing cortisol levels [198].

### 4.6. Glucocorticoid-Induced Cellular Senescence in Skeletal Damage

Skeletal damage is a common metabolic disorder that is frequently secondary to Cushing’s syndrome. Abnormal cortisol levels in patients with Cushing’s syndrome can induce osteopenia in 40–78%, osteoporosis in 22–57% and skeletal fractures in 11–76% of patients [199]. Glucocorticoids can suppress the maturation, proliferation and function of osteoblasts, eventually resulting in bone loss [200]. An in vitro study demonstrated that both p53 and its downstream p21 were reported to be stimulated under the treatment of dexamethasone, which leads to G1 cell cycle arrest of murine osteoblastic cell lines [18]. This senescent phenotype could be attenuated by the silencing of the GR gene [18]. In fetal mice, prenatal dexamethasone exposure induced tardive skeletal development via increased activity of SA-β-GAL and suppressed Ki-67 expression [201]. In addition, the clearance of senescent cells by Dasatinib and quercetin alleviated dexamethasone-induced senescent bone tissue with increased bone mineral density and decreased SA-β-GAL activity in mouse models [201,202]. In mice treated with prednisolone, increased expression of p16 and SA-β-GAL was detected, which resulted in a senescent phenotype through the DPP4-GLP-1 pathway [203]. This senescent phenotype could be rescued by treatment with Dasatinib and Quercetin and the anti-inflammatory drug Ruxolitinib [203]. In patients with congenital adrenal hyperplasia, the higher bone mineral density was associated with the lower circulating cortisol level due to the deficiency of 21-hydroxylase, although its detailed mechanism remains to be clarified [204].

### 4.7. Adrenal Androgen-Induced Cellular Senescence

Adrenal androgen is more actively present in young or fetal humans and primates rather than certain common experimental rodents, such as mice and rats. Therefore, due to this specific characteristic, senescent phenotypes induced by adrenal androgens, including DHEA and DHEAS, have rarely been reported. DHEA rather than DHEAS was reported to inhibit the proliferation of human umbilical vein endothelial cells, inducing cell cycle arrest in the G1 phase via the phosphorylation of the RB protein and increased expression of p53 and p21 [205]. Its derivatives, such as 17β-estradiol and testosterone, were not identified to play an antiproliferation role in umbilical vein endothelial cells, indicating that the antiproliferation was only caused by their precursor DHEA, but not secondary to DHEA-converted steroids [205]. In rabbit vascular smooth muscle cells, DHEAS induced an antiproliferative effect via increased p16 expression rather than p53 or p21 expression [206]. DHEA was also reported to inhibit the proliferation of rat mammary tumors through the activation of p16, p21 and SA-β-GAL in a p53-independent manner and eventually induced cell cycle arrest in the G1 phase.

## 5. Conclusions

This is the first study to systemically review the role of cellular senescence in adrenal biology. Along with aging, the adrenal structure and function gradually change and sometimes become pathological. In older individuals, adrenal glands were reported to become atrophied, resulting in lower aldosterone and adrenal androgen levels but higher cortisol levels. The detailed mechanism remains unknown, although cellular senescence is regarded to play an essential role in age-dependent alternations of adrenal glands. In aldosterone-producing cells, the senescent phenotype may impede the aldosterone-producing ability in both normal adrenal glands and APAs. In addition, the senescent phenotype in CPAs does not negatively impact hormonal production; on the contrary, it may even stimulate cortisol secretion. In ZR cells and adrenal androgen-producing adenomas, cellular senescence has rarely been explored, although they harbor much lipofuscin. In ACCs, oncogene-induced senescence was identified, which compensatorily followed carcinogenesis or tumor progress. Based on the different cellular subtypes, compact cells of both APA and CPA present a more senescent phenotype than clear cells, especially in those with somatic mutations. Adrenocortical steroids including aldosterone, cortisol and adrenal androgen also induce a periphery senescence in many tissues. Many tissues, such as those of the kidneys, brain, liver, bone, colon, vascular smooth muscle and lungs, were demonstrated to lose their proliferation and be senescent under the physiological or pathophysiological range of aldosterone or cortisol levels.

## Data Availability

Data sharing not applicable.
